# The Influence of the Physical-Mechanical Parameters of Rock on the Extent of the Initial Failure Zone under the Action of an Undercut Anchor

**DOI:** 10.3390/ma14081841

**Published:** 2021-04-08

**Authors:** Józef Jonak, Robert Karpiński, Andrzej Wójcik, Michał Siegmund

**Affiliations:** 1 Department of Machine Design and Mechatronics, Faculty of Mechanical Engineering, Lublin University of Technology, Nadbystrzycka 36, 20-618 Lublin, Poland; a.wojcik@pollub.pl; 2 KOMAG Institute of Mining Technology, Pszczyńska 37, 44-100 Gliwice, Poland; msiegmund@komag.eu

**Keywords:** physical and mechanical parameters of rocks, mining extracting tool, undercut anchor, FEM analysis, rock breakout failure, rock cone failure

## Abstract

This paper presents the results of a numerical FEM (Finite Element Method) simulation of the formation of a rock failure zone in its initial stage of development. The influence of rock parameters, such as the Young’s modulus, Poisson’s ratio and friction factor of the rock in the contact zone with the working surface of the undercut anchor head, were taken into account. The obtained results of FEM simulations were compared with the results of field tests conducted in Polish mining plants extracting rock raw materials.

## 1. Introduction

**Figure 1:**
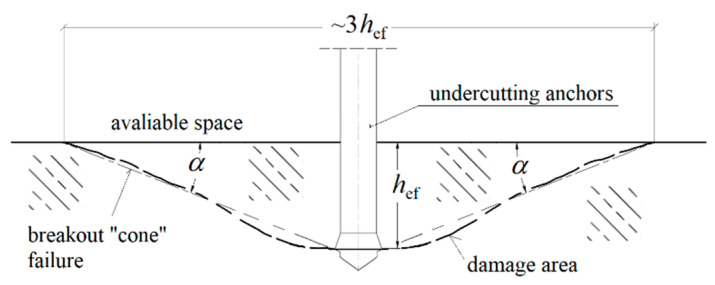
Breakout “cone” failure in rock materials (*h*_ef_—effective anchoring depth, *α*—the angle of the cone of destruction of the medium).

Numerical modelling [1,2,3], together with experiments [4,5,6,7], enables a detailed understanding of the actual behavior of structures and their optimization. Typically, this type of anchor is used mainly in the fastening of steel structural elements in concrete engineering structures (e.g., [8,9,10]). There are many varieties of anchoring elements, among them the undercut anchor, which is characterized by relatively simple installation and high anchor pull-out force. Among the important issues that are addressed in the topic of anchoring, one can distinguish the problem of estimating the load capacity of anchors (working alone as well as in groups) depending on the effective anchorage depth (*h*_ef_), the strength of concrete, the distance from the edge of the object, the ratio of the anchorage depth to the thickness of the concrete element in which the anchor is embedded [1] or the interaction of the so-called concrete failure cones in multi-anchor systems [4,11,12]. In the characterization of the extent of the damage zone, according to the concrete capacity design method) [13,14], the damage zone is usually approximated by a cone defined by the value of the so-called damage cone angle alfa (Figure 1).

According to several studies [12,15], the value of the failure cone angle depends mainly on the effective anchorage depth *h*_ef_, the anchor head diameter and the angle of the undercutting head.

Numerical analyses of the process of material structure failure under the action of anchors of various designs show a significant influence of physical and mechanical parameters of the medium (mainly concrete). Among the parameters considered, the influence of Young’s modulus, Poisson’s ratio or coefficient of friction in the contact between the head and the medium are mainly emphasized (in addition to compressive or tensile strength) [11,16].

Due to the limited knowledge in the field of pull-out of anchors installed in rock media, research has been undertaken (e.g., [17,18,19]) to find out whether previous findings on the propagation of concrete failure under the action of undercut anchors can be transposed to processes occurring in natural rock media. In comparison to concrete, such media have a complex internal structure (e.g., layering), as well as more varied physical and mechanical parameters, related, among other things, to the history of their formation or location in the rock mass. In view of the above, an attempt to develop an atypical method of rock dislodgement by pulling out undercut anchors [17,18,19] requires research and analysis, broadening the state of knowledge, in the field of mechanics of rock failure under the action of undercut anchors.

The motivation to take up the subject were the results of field tests (e.g., [18]), where during pulling out of undercutting anchors installed in rock masses, the values of pull-out forces (load capacity of anchors) and the range of failure zone were found to be significantly different from those resulting from the current state of knowledge (until now, the subject of load capacity of anchors concerned concrete structures). Numerical FEM (Finite Element Method) studies performed so far by the authors of this paper have shown significant differences in the progress and extent of failure zone in concrete and rock [6,17]. The limitations of the ABAQUS algorithms used to determine the trajectory of fractures in the covered state were subject to high error rates [19,20,21]. This was especially true for the determination of the instantaneous direction of crack propagation at this stage of development. The course and extent of the failure zone of the rock medium under the action of undercutting anchors is of particular interest to the authors because of the need to develop an effective method for tracking the potential interaction of the failure zones of the rock medium in systems of multiple anchors, as well as sequential pulling of anchors. This is a very important issue in the installation of infrastructure elements under mining conditions.

## 2. Materials and Methods

The main focus of the present study was to find out the influence of parameters of the rock medium such as the value of Young’s modulus and Poisson’s ratio, on the formation of the extent of the failure zone in the initial phase of failure. This is due to the fact that in industrial conditions, there is often a difficulty in determining the material data used in the non-linear models available in FEM numerical analysis programs (Finite Element Method). The use of non-linear models is planned in subsequent analyses and the results will be presented in subsequent papers.

**Figure 2:**
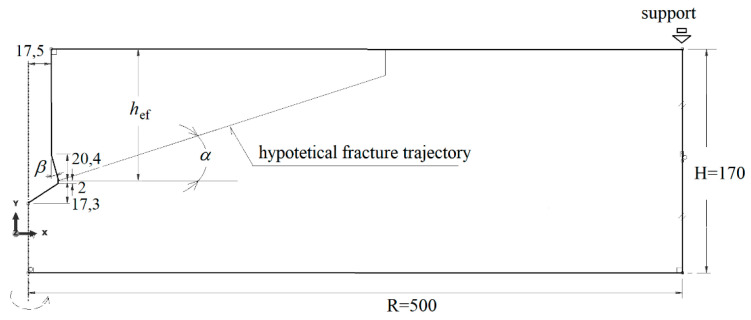
Axisymmetric model of anchor interaction with rock: *β*—anchor head angle, *α*—angle of the potential failure cone.

The analysis was conducted using the FEM program–ABAQUS [22] (Abaqus 2019, Dassault Systemes Simulia Corporation, Velizy Villacoublay, France), the XFEM algorithm was applied. The initial angle of penetration of the fracture surface was analyzed, as it determines the size (volume) of the rock mass peeled off in the process of controlled experimental pull-out of undercut anchors. For the purposes of the analysis, an axially symmetric model of the influence of the undercut anchor on the rock was adopted, as shown in Figure 2. The model was assumed to have a radius of *R* = 500 mm and a depth of *H* = 170 mm. The anchoring depth is assumed to be *h*_ef_ = 100 mm. As can be seen from the model dimensions, the ratio of the radius of the boundary conditions (three supports evenly distributed over the assumed diameter) of the anchor pull-out device *R* to the effective anchorage depth *h*_ef_, i.e., *R*/*h*_ef_ was 5. Such a ratio value, in the light of CCD—cone capacity design method) [13] is sufficient to prevent the potential effect of the boundary restraints on the distribution of stresses in the zone of formation of the “cone of failure”. According to the CCD method, the diameter of the base of the failure cone measured at the concrete surface is approximately 3*h*_ef_ (Figure 1). The dimensions of the rock undercut, representing the outline of the anchor after installation (after rock undercut), are shown in Figure 2. The height of the conical part of the head was assumed to be 20.4 mm, the diameter of the simulated hole was *Φ* = 2 × 17.5 mm = 35 mm (which corresponds to the recommended hole diameter for a Hilti anchor of M20 mm diameter). Head angle *β* was assumed to be 20°.

**Figure 3:**
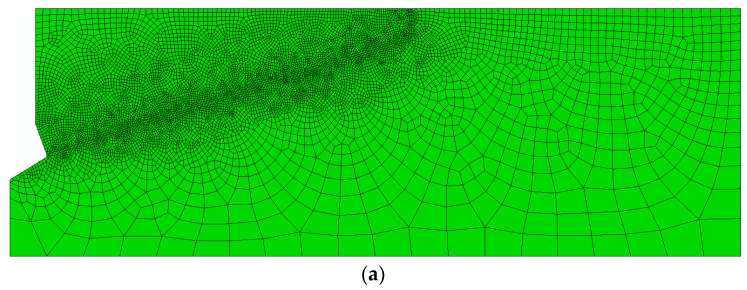

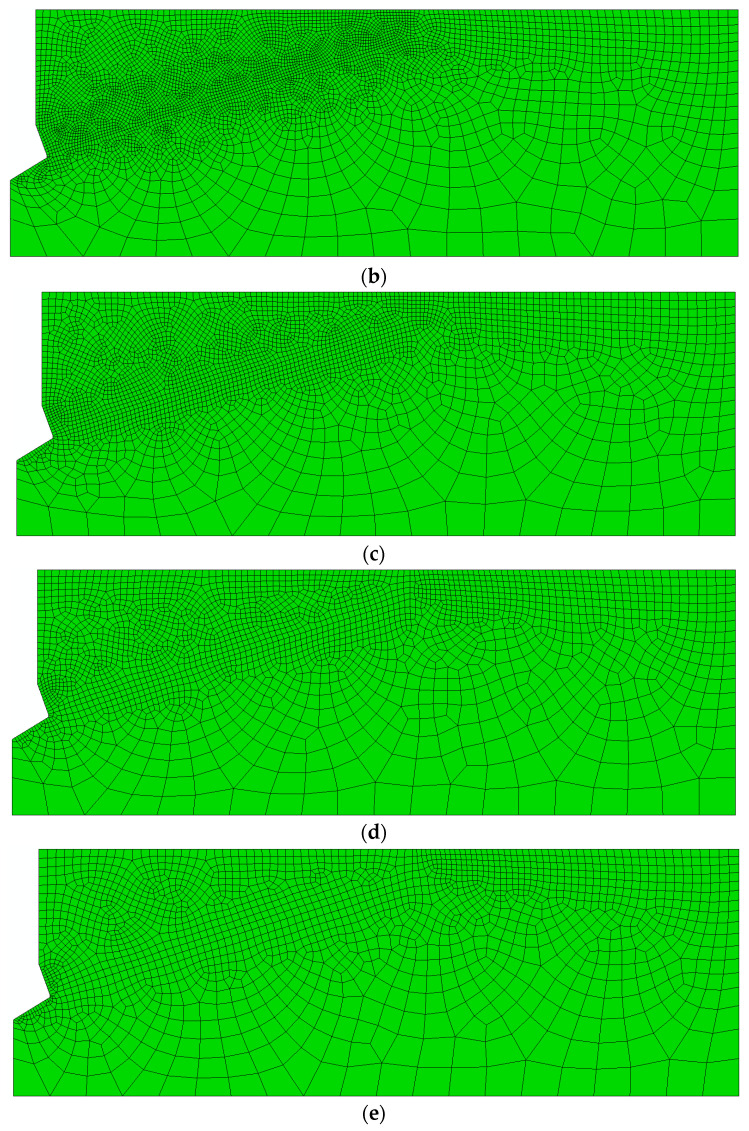
Finite element mesh of the model with compaction in the area of the hypothetical propagation of the “cone” of destruction. The different densities of the mesh along the expected crack line: (**a**) 1 mm, (**b**) 2 mm, (**c**) 3 mm, (**d**) 4 mm, and (**e**) 5 mm.

The interaction of the conical head surface with the rock was treated as a contact issue. Surface-to-surface contact between the rock and the anchor on the conical and upper cylindrical surfaces was assumed. The “Penalty” friction option was used in ABAQUS program. With the automatic finite element mesh generator mode of the program, the mesh shown in Figure 3 was obtained. CAX4R planar elements were used: axisymmetric and linear (two-parameter linear function) (four nodes) with hourglass. In the region of the predicted crack surface propagation (based on existing fracture penetration models existing in the subject literature) (Figure 2), mesh refining was introduced, as can be seen in Figure 3. The authors decided to examine the dependence of the results on the mesh size. For this purpose, five variants were modeled (for other fixed parameters) but with different densities of the mesh along the expected crack line: 1 mm, 2 mm, 3 mm, 4 mm and 5 mm.

**Figure 4:**
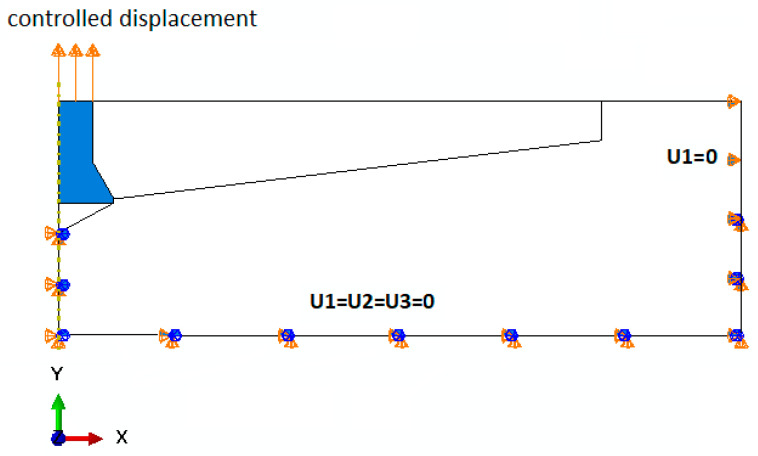
Model boundary conditions.

The restraints to the nodes of the finite element mesh of the model (Figure 4) were made in the axis of the model (under the anchor) and along the bottom (horizontal) and outermost sides of the model. For the most part, the restraints were stripped of all degrees of freedom (U1 = U2 = U3 = 0).

The anchor was given a controlled displacement, vertically upwards, along the anchor axis (the *Y* axis of the model).

### Simulation Assumptions

**Rock Material**

Sandstone: elastic, isotropic, with the following parameters:

- Young’s modulus E_I_ = 14276 MPa and Poisson’s Ratio *ν*_1_ = 0.15 and *ν*_2_ = 0.20
- Young’s modulus E_II_ = 9287MPa and Poisson’s Ratio *ν*_3_ = 0.25 and *ν*_4_ = 0.30
- Tensile strength (two options) *f*_tI_ = 7.74 MPa, *f*_tII_ =15MPa

Selection of the physical and mechanical parameters of the rock medium was based on our own research [20,21], as well as information contained, for example, in [23,24]. The coefficient of friction in the contact between the steel anchor and the rock medium, according to various literature data, is assumed to be 0.35–0.65 and most commonly 0.4–0.5 on average (e.g., [25]). In addition, for comparison purposes, a simulation was performed for Coulomb friction coefficients in the anchor–rock contact zone, equal to *μ*_1_ = 0.2 and *μ*_2_ = 0.4. The friction coefficient values of metal anchors in contact with sandstone are difficult to estimate under field test conditions. The values adopted for the simulation were based on a number of literature sources on aspects of fixing anchors, as well as mining tools. The authors’ experience shows that for very resistant sandstones with large grain diameters the coefficient of friction between the rock and the mining tool material, including steel, may oscillate in the range of 0.1–0.2. Therefore, the simulation assumed a coefficient of friction equal to 0.2, which will characterize compact rocks of high strength (e.g., red sandstones) and equal to 0.4, adequate for a group of rocks of low strength and fine grain size (e.g., grey sandstones).

**Undercut anchors**—steel: elastic, isotropic, elastic modulus—*E* = 210,000 MPa,

Poisson’s Ratio—*ν* 0.3.

Conditions for the initiation and development of failure:

Damage initiation in rock material: maximal principal stress (two options): *f*_tI_ = 7.74 MPa, *f*_tII_ = 15 MPa

Damage evolution: type: energy, softening: linear. Damage for traction separation laws: maximal principal stress damage, fracture energy = 0.335 N/mm.

**Figure 5:**
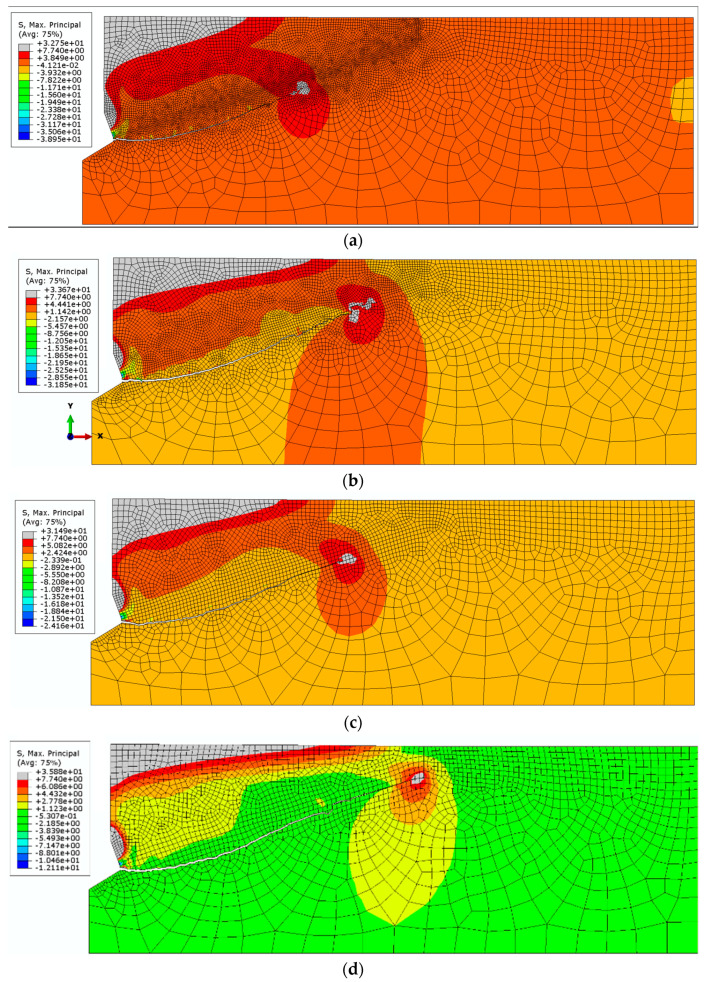

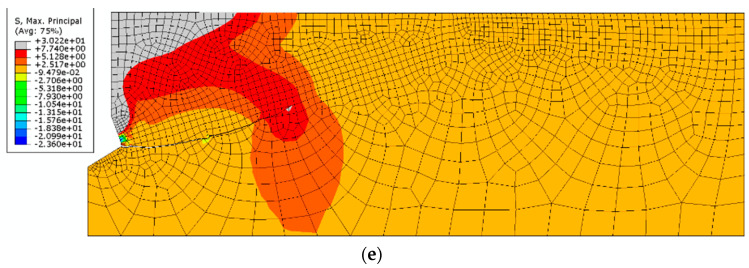
Comparison of the crack path shape for various mesh densities. (**a**) the smallest mesh size = 1 mm, (**b**) 2 mm, (**c**) 3 mm, (**d**) 4 mm, and (**e**) 5 mm.

It can be verified that the dimensions of the sides of the element with a 2-mm or 3-mm have a disturbance in the stress distribution around the crack tip (Figure 5). On the other hand, the tests for a 1-mm mesh were discontinued because the simulation progress was stalled for a long time. The tests for 4- and 5-mm meshes show a large fluctuation in slit propagation. The conclusion is that the choice of rafting is construction, but the difference between the final and the smallest construction is small. Moreover, the fracture lines in the initial propagation are complementary parts, which can be seen in Figure 5. The fact that the size of the FEM mesh is of negligible importance in the X-FEM method is consistent with the literature [26].

As a result, a model with an element side dimension of 2 mm was selected for further analysis.

**Figure 6:**
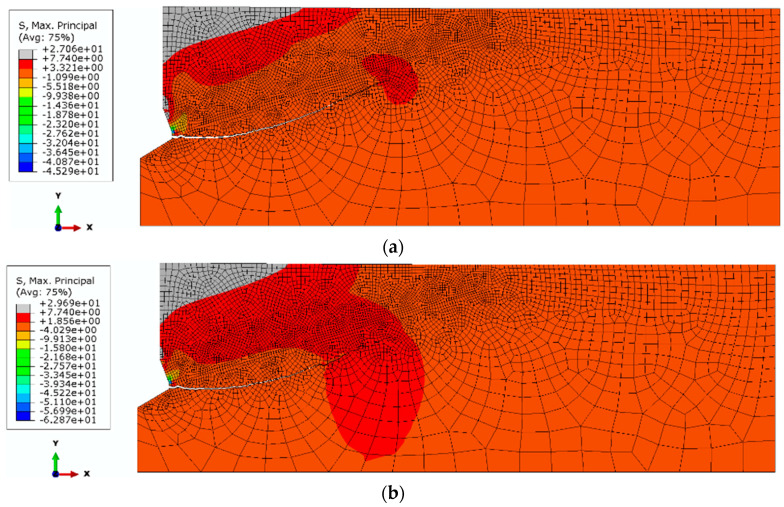
Initial course of damage propagation and distribution of maximum stresses for: E_II_ = 9287 MPa, *μ* = 0.4; *β* = 20° and Poisson’s Ratio, (**a**) *ν*_3_ = 0.25; (**b**) *ν*_4_ = 0.30.

**Figure 7:**
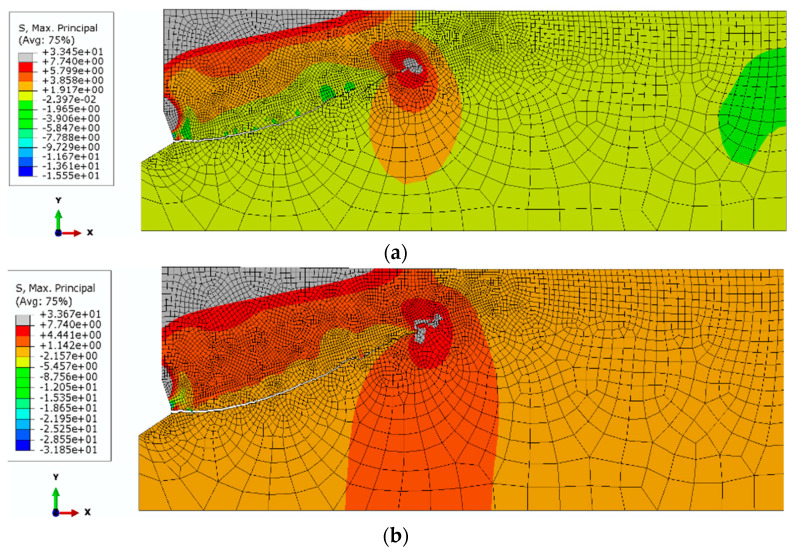
The course of the damage propagation and the distribution of maximum scheme is 14,276 MPa, *μ* = 0.4; *β* = 20° and Poisson’s Ratio, (**a**) *ν*_1_ = 0.15; (**b**) *ν*_2_ = 0.20.

**Figure 8:**
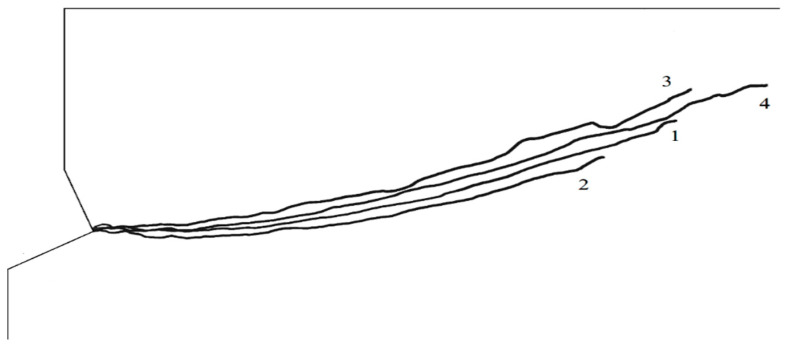
Summary of the determined damage trajectories: No. 1—E_II_ = 9287 MPa, *ν*_3_ = 0.25; No. 2—E_II_ = 9287 MPa, *ν*_4_ = 0.30; No. 3—E_I_ = 14,276 MPa, *ν*_1_ = 0.15, No. 4—E_I_ = 14,276 MPa, *ν*_1_ = 0.20.

In the initial stage of the analysis, a constant value of the Coulomb friction coefficient was assumed to be equal to *μ*_2_ = 0.4. The value of the head cone angle was constant and equaled *β* = 20°. The results of the simulations carried out are illustrated in Figure 6, Figure 7 and Figure 8.

It can be seen from Figure 6 that an increase in Poisson’s ratio from *ν* = 0.25 to 0.30 results in a decrease in the initial angle of propagation of the failure of the medium (α, Figure 1 and Figure 2). The amount of deformation of the material perpendicular to the axis of the anchor head increases (towards the *OX* axis, Figure 7); the crack penetrates more intensively into the material (Figure 7b).

Trajectories No. 3 and No. 4, as well as No. 1 and No. 2, show a clear effect of Young’s modulus on the propagation of the failure zone. In general, the higher the value of the modulus, the lower the penetration of the trajectory into the material. The change of Poisson’s ratio additionally differentiates the obtained results. It can be seen from the comparison that an increase in Poisson’s ratio generally favors an increase in the extent of the damage zone and an increase in penetration of the damage zone (initial value of the angle *α*).

Comparison of curves 2 and 3 clearly highlights the influence of the value of Young’s modulus. A higher value of Young’s modulus (run 3) results in a significantly lower penetration depth than in the case of lower values (run 2).

**Figure 9:**
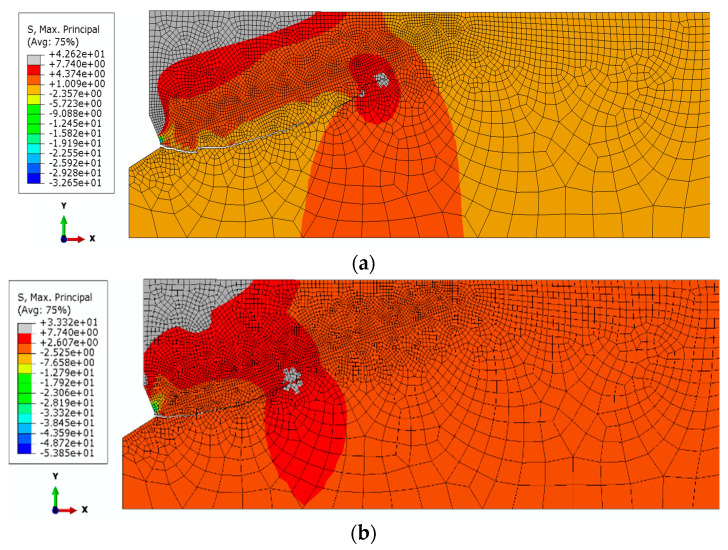
The course of the damage propagation and the distribution of maximum stresses: elastic modulus—E_I_ = 14,276 MPa, Poisson’s ratio *ν* = 0.25, β = 25°, (**a**) *μ*_1_ = 0.2, (**b**) *μ*_2_ = 0.4.

**Figure 10:**
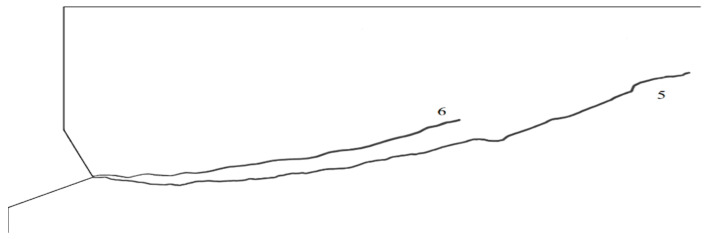
Comparison of destruction trajectories: elastic modulus—E_I_ = 14,276 MPa, Poisson’s ratio *ν*_3_ = 0.2. Trajectory No. 5—coefficient of friction *μ*_1_ = 0.2, No. 6—coefficient of friction *μ*_2_ = 0.4.

To extend the scope of the simulations, in order to investigate the influence of the friction coefficient on the propagation of the rock failure zone, the next stage of the analysis assumed elastic modulus—E_I_ = 14,276 MPa and Poisson s ratio *ν*_3_ = 0.25. Simulations were performed assuming the friction coefficients *μ*_1_ = 0.2 and *μ*_2_ = 0.4. The simulation results are illustrated in Figure 9 and Figure 10.

It is clear from Figure 9b that an increase in the friction coefficient limits the deep penetration of the failure zone. The initial value of the angle of the failure “cone” clearly increases. This relationship is illustrated more clearly in Figure 10, where both trajectories are superimposed on a single graph.

Run 5 (Figure 10), for material data—E_I_ = 14,276 MPa, Poisson’s ratio *ν*_3_ = 0.2 and friction factor *μ* = 0.2, penetrates much deeper into the material in the initial stage and has a much greater range along the *OX* axis of the coordinate system. Run 6 (Figure 10), for material data—E_I_ = 14,276 MPa, Poisson’s ratio *ν*_3_ = 0.2 and for friction factor *μ* = 0.4 has a smaller range and penetrates less deeply into the material than run 5.

**Figure 11:**
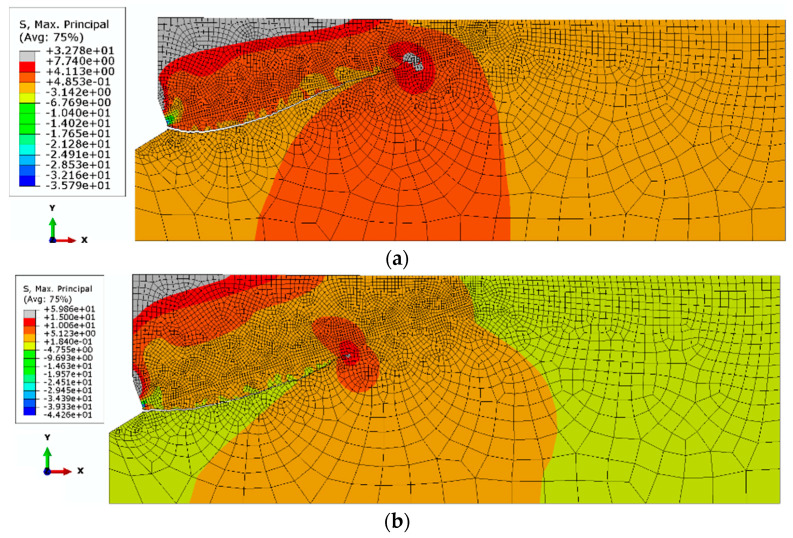
The course of the trajectory of failure in the initial stage of material fracture, for various variants of tensile strength *f*_t_: (**a**) *f*_tI_ = 7.74 MPa, (**b**) *f*_tII_ = 15 MPa.

The analysis showed that in the considered range of parameters, the influence of the compressive strength *f*_t_ on the course of the damage trajectory in the initial propagation phase is clear, as shown in Figure 11. The fracture trajectories are clearly different. For weaker sandstones, the propagation of destruction in the initial stage is clearly negative with the propagation angle (*α*). At a later stage of destruction development, there is a noticeable deflection of the trajectory, which will lead to an increase in the extent of disengagement. For stronger sandstones, the destruction is initiated at a positive angle and progresses towards the free surface. This limits the extent of dislocation.

## 3. Discussion

The results obtained from the numerical tests are consistent in terms of trends with those observed during load capacity tests of anchors installed in concrete, e.g., [16]. Physical parameters of the medium, such as Young’s modulus (E), Poisson’s ratio (*ν*) and Coulomb’s contact friction coefficient (*μ*), have a significant impact on the mode of failure of the rock medium. However, the influence varies. The most pronounced influence is observed for the Young’s modulus and the coefficient of friction (*μ*). A less pronounced influence is observed for the Poisson’s ratio (*ν*). For weaker sandstones, the propagation of destruction in the initial stage is clearly negative with the propagation angle (*α*). At a later stage of the destruction development, there is a noticeable deflection of the trajectory, which will lead to an increase in the extent of disengagement. For stronger sandstones, the destruction is initiated at a positive angle and progresses towards the free surface.

## 4. Validation of Numerical Results

**Figure 12:**
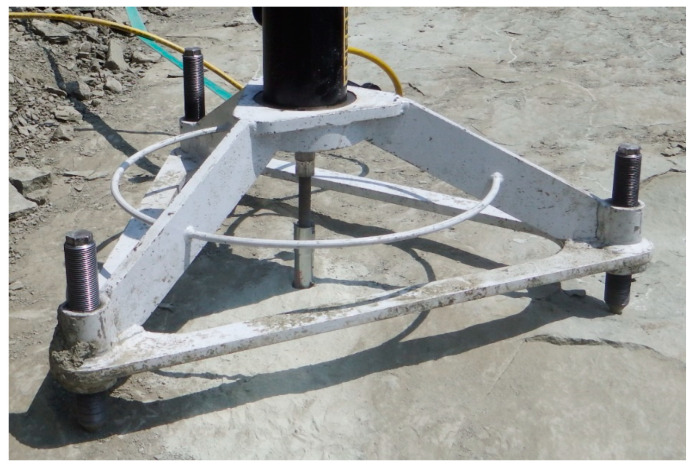
Device for pulling anchors.

Extensive field studies carried out within the framework of the RODEST project [17,27] and ongoing analyses on the possibility of using the process of pulling out undercut anchors installed in the rock medium, [17,18] as an alternative stripping technology, have revealed the influence of a much larger number of factors (related to the current state of the rock in the rock mass) than is the case with concrete. An anchor pull-out device with three adjustable supports spaced at a radius of *R* = 500 mm was used (Figure 12).

The maximum anchorage depth resulted from the strength of the anchor material (steel) and was *h*_ef max_ = 140 mm. For the purposes of this analysis, results were selected from *h*_ef_ = 100 mm. The research was conducted, among others, at the “Brenna” and “Braciszów” mining sites [17,19,27]. The anchors were located in homogeneous rock media, without clear cracks and structure disturbances.

Physical and mechanical parameters of the studied rocks (sandstones) are summarized in Table 1. The friction coefficient *μ* was not determined. However, practice shows that due to varied moisture content of rocks and varied petrographic structure of sandstones, friction coefficients may significantly vary, e.g., [23,24]. For mining tools, these values are also adopted at varying levels (e.g., [8,28]), as in some issues of fixing designs using anchors [4].

**Figure 13:**
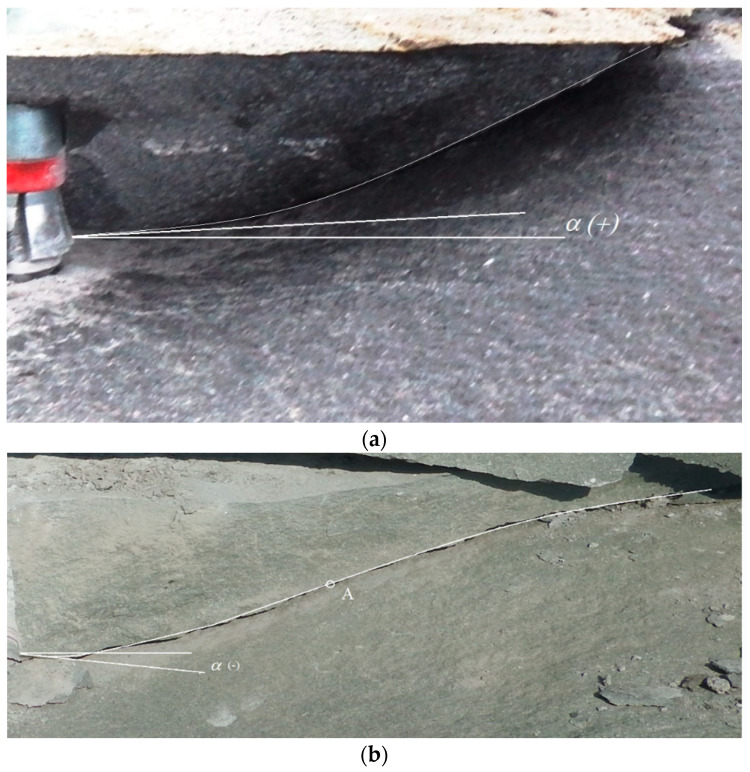
Failure trajectories of the rock medium: (**a**) Braciszów sandstone and (**b**) Brenna sandstone; *α*—initial angle of failure trajectory, A—inflection point of the failure trajectory.

It was observed that in the case of “stronger” sandstones (e.g., from the Braciszów mine) the failure trajectory has a different character than in the case of “weak” sandstones, e.g., from the Brenna mine (Figure 13).

The trajectory of failure of the “stronger” sandstones (Braciszów) is clearly close to a parabola (Figure 13a). The initial angle of the failure trajectory *α* generally takes the values >0°. In weak sandstones (Brenna type), on the other hand, the initial trajectory is also close to a parabola, but the initial trajectory angle *α* often takes values smaller than 0°. In addition, as failure develops (point A, Figure 13b), there is a clear change in the failure trajectory. From this point on, there is a pronounced flattening of the trajectory, favoring large ranges of detachments on the free rock surface.

The trends in sandstone failure trajectories observed during the field investigations generally confirm the results of the numerical analyses. The authors are aware that in real rock media, simultaneous coincidence of the influence of all considered factors may occur and the interpretation of the results may be considerably difficult. Further in-depth research on the subject is needed in order to improve knowledge and provide more precise conclusions.

Experimental tests have positively verified the results of numerical tests, especially in the context of the influence of compressive strength on the course and extent of the rock destruction zone under the action of the undercutting anchor.

## 5. Conclusions

Numerical analyses and field investigations have shown that in the process of rock structure failure by undercut anchors, the initial angle of failure propagation and the extent of failure (detachment) depends significantly on the examined physical and mechanical parameters of the medium, such as the Young’s modulus (E), the Poisson’s ratio (*ν*) or the friction coefficient in the contact between the tool and rock (*μ*) and the compressive strength *f*_t_. The most pronounced effects were observed for the friction coefficient and Young’s modulus. The influence of other factors influencing the course and extent of failure of the rock medium (e.g., depth of anchorage, angle of the conical undercutting head, breaking strength or angle of internal friction of the rock) has been successively studied (e.g., [19,20,21]) and will be the subject of further analyses.

The obtained results extend the knowledge gained during the analysis of the impact of undercutting anchors on the rock medium conducted earlier by the team of authors of this study, using the “cohesive zone” [6] and 3D models and ABAQUS program [19,20,21].

## Figures and Tables

**Table 1:** Physical and mechanical parameters of the studied rocks.

Rock	*f*_c_ (MPa)	*f*_t_ (MPa)	*E* (MPa)	*ν* (–)	*K*_IC_ (N/mm^3/2^)	*G*_IC_ (N/m)	*c* (MPa)	Description
“Braciszów” sandstone	187.232	7.614	15.745	0.203	69.184	303.995	14.5	Sandstone strong, compact
“Brenna” sandstone	95.562	3.209	13.727	0.148	25.655	47.946	6.0	Sandstone layered, weak

*f*_c_—compressive strength, *f*_t_—tensile strength, *c*—cohesion, *E*—Young’s modulus, ν—Poisson’s ratio, *K*_IC_—critical stress intensity factor, *G*_IC_—critical strain energy.

## Data Availability

The data presented in this study are available on request from the corresponding author.
